# Rosemary (*Rosmarinus officinalis* L., syn *Salvia rosmarinus* Spenn.) and Its Topical Applications: A Review

**DOI:** 10.3390/plants9050651

**Published:** 2020-05-21

**Authors:** Lucas Malvezzi de Macedo, Érica Mendes dos Santos, Lucas Militão, Louise Lacalendola Tundisi, Janaína Artem Ataide, Eliana Barbosa Souto, Priscila Gava Mazzola

**Affiliations:** 1 School of Medical Sciences, University of Campinas (Unicamp), Campinas 13083-894, Brazil; lucas.mmalvezzi@gmail.com; 2 Institute of Biology, University of Campinas (Unicamp), Campinas 13083-862, Brazil; ericamendes_s@hotmail.com; 3 Faculty of Pharmaceutical Sciences, University of Campinas (Unicamp), Campinas 13083-871, Brazil; lucxsmilitao@gmail.com (L.M.); tundisi.l@gmail.com (L.L.T.); 4 Department of Pharmaceutical Technology, Faculty of Pharmacy, University of Coimbra (FFUC), 3000-548 Coimbra, Portugal; souto.eliana@gmail.com; 5 CEB-Centre of Biological Engineering, University of Minho, Campus de Gualtar, 4710-057 Braga, Portugal

**Keywords:** rosemary, antioxidant, anti-inflammatory, flavonoids, polyphenols, terpenes

## Abstract

Topical application is an important administration route for drugs requiring local action on the skin, thereby avoiding their systemic absorption and adverse side effects. *Rosmarinus officinalis* L. (syn. *Salvia rosmarinus* Spenn.), popularly known as rosemary, is an aromatic plant with needle-like leaves belonging to the *Lamiaceae* family. Rosemary has therapeutic properties and has been used in the folk medicine, pharmaceutical, and cosmetics industries, mainly for its antioxidant and anti-inflammatory properties, which are attributed to the presence of carnosol/carnosic and ursolic acids. The therapeutic use of rosemary has been explored for the treatment of inflammatory diseases; however, other uses have been studied, such as wound healing and skin cancer and mycoses treatments, among others. Besides it therapeutic uses, rosemary has potential applications in cosmetic formulations and in the treatment of pathological and non-pathological conditions, such as cellulite, alopecia, ultraviolet damage, and aging. This review aims to critically discuss the topical applications of rosemary found in the literature while also offering relevant information for the development of topical formulations of its bioactive compounds.

## 1. Introduction

The use of herbal drugs to treat a broad spectrum of diseases and/or to modify non-pathological states [1,2,3,4] has increased worldwide. It is known that the secondary metabolites of plants have therapeutic effects; many have been used in the treatment of different diseases, such as obesity [5] and brain [6] and skin diseases [7] as well as in the treatment of non-pathological states, such as aging [8].

*Rosmarinus officinalis* L., commonly known as rosemary, belongs to the *Lamiaceae* family. The genus Rosmarinus has been merged into the genus Salvia in a recent phylogenetic analysis. This means that the *Rosmarinus officinalis* is no longer the correct name of the species studied. Since the name *Salvia officinalis* was already occupied when the merger was done, this species needed a new specific epithet in Salvia, so it is now known under the name *Salvia Rosmarinus* [9,10,11]. It is an aromatic plant with needle-like leaves that is cultivated worldwide. Rosemary has therapeutic properties and has been used in folk medicine as an oral preparation to relieve renal colic, dysmenorrhea, and muscle spasms [12,13,14]. Rosemary has antifungal, antiviral, antibacterial, anti-inflammatory, antitumor, antithrombotic, antinociceptive, antidepressant, antiulcerogenic, and antioxidant activities [13,14,15]. Several medicinal applications for *R. officinalis* have been identified, such as treatment of disorders associated with the nervous, cardiovascular, gastrointestinal, genitourinary, menstrual, hepatic, and reproductive systems and with respiratory and skin conditions [13]. Owing to its diverse properties, rosemary has also been used widely in the food and cosmetics industries [16].

Many biomolecules have been identified to be responsible for the biological effects of rosemary essential oil and crude extract. However, specific compounds causing these effects have rarely been identified; this is due to the synergistic actions of several metabolites present in rosemary [17]. Therefore, it is difficult to associate a therapeutic or cosmetic activity with an isolated biomolecule. del Baño et al. characterized the distribution of rosemary flavonoids (eriocitrin, luteolin 3’-O-β-D-glucuronide, hesperidin, diosmin, isoscutellarein 7-O-glucoside, hispidulin 7-O-glucoside, and genkwanin) in the leaves, flowers, roots, and stems during different stages of the plant’s growth [18]. It was also reported a high concentration of flavonoids, polyphenols, and terpenes in *R. officinalis* leaves [19]. Rosemary contains an abundance of secondary metabolites, and their identification by ultra- and high-performance liquid chromatography and gas chromatography has revealed high contents of profile phenolic compounds (diterpenoids and flavonoids) and volatile compounds [20,21].

The biological activities of secondary metabolites and extracts of *R. officinalis* were reported in studies investigating various effects such as its antitumor, antioxidant, anti-infectious, anti-inflammatory, and analgesic activities and effects on the central nervous system, endocrine system, disorders such as cardiac remodeling after myocardial infarction, body weight changes, dyslipidemia, cerebral ischemia, hepato-nephrotoxicity, stress, and anxiety [22,23]. The anti-inflammatory activity of rosemary has been attributed to the presence of carnosol and carnosic, rosmarinic, ursolic, oleanolic, and micromeric acids, which act synergistically [24,25,26]. Specifically, the anti-inflammatory effect was also attributed to the synergic effects of ursolic and micromeric acids present in rosemary extract. The attribution of anti-inflammatory effects of the *R. officinalis* extract was due to the presence of ursolic, oleanolic, and micromeric acid acting in combination [24].

The skin is the largest organ in the human body; sensation, regulation, and protection are among its most critical functions [27]. To enhance the permeation of bioactive compounds through the skin, many approaches have been proposed. Of these approaches, nanocarriers including nanoemulsions, lipid nanoparticles, and liposomes have become popular owing to their lipid composition, enhanced biocompatibility, and biodegradability [28,29,30,31,32]. The release profile of the loaded bioactive compound can be modulated by altering the physicochemical composition of the nanocarrier matrix [33].

The aim of this review was to survey the publications related to the topical applications of rosemary and to discuss the formulations available for the delivery of the secondary metabolites of *R. officinalis*.

## 2. Methods

The Web of Science, Google Scholar, and SciELO databases were selected for research on the topical uses of rosemary, using “*Rosmarinus officinalis*” and “rosemary” as keywords. The authors are aware of the nomenclature update; however, literature still shows *Rosmarinus officinalis* as the main name for rosemary.

## 3. Pharmaceutical Activities

**Figure 1:**
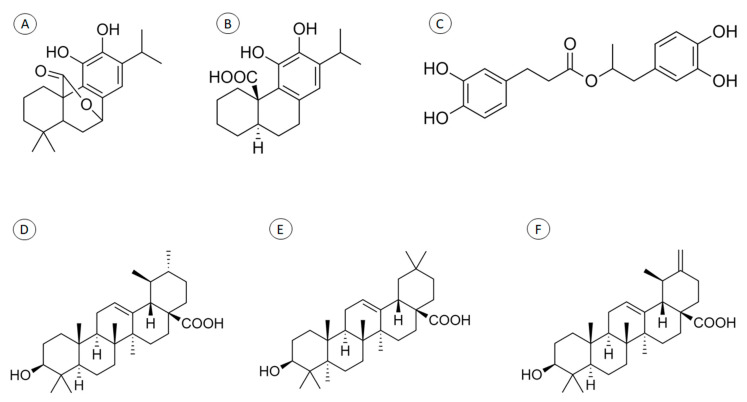
Chemical structure of some Rosmarinus officinalis secondary metabolites: carnosol (**A**), carnosic acid (**B**), rosmarinic acid (**C**), ursolic acid (**D**), oleanolic acid (**E**), and micromeric acid (**F**).

Rosemary (Table 1) has attracted attention because it contains secondary metabolites with therapeutic potential, such as carnosol and carnosic, rosmarinic, ursolic, oleanolic, and micromeric acids (Figure 1). These compounds have been applied topically and studied for their anti-inflammatory capacity, wound-healing potential, effects on tissue survival, anti-skin-cancer effects, antinociceptive effects, antifungal effects, and UV-protective activity. The triterpenes ursolic, oleanolic, and micromeric acids exhibit the strongest anti-inflammatory activity of all the secondary metabolites [24]. In addition to the gross extracts, it is possible to use rosemary essential oil for topical applications [32]. The major constituents of the oil are β-pinene, 1, 8-cineole, borneol, camphor, limonene, and verbenone [34]. 

### 3.1. Anti-inflammatory Activity

The inflammatory activity of *R. officinalis* extract is attributed to the presence of carnosol and carnosic acid [53] and of ursolic, oleanolic, and micromeric acids [24].

The bioactive compound carnosic acid was reported to be a potent nitric oxide (NO) inhibitor; NO is a pro-inflammatory mediator that induces or enhances the inflammatory process [35]. Low concentrations of this metabolite (6.2 μg/mL) inhibited NO by approximately 72%, whereas complete inhibition was reported at concentrations of >12.5 μg/mL [36]. Using the 2,2-diphenyl-1-picryl-hydrazyl-hydrate (DPPH) assay, the *R. officinalis* extract was shown to possess strong antioxidant activity, supporting its potential as an anti-inflammatory agent. The extract also exhibited antiplatelet activity, which is instrumental for the improvement of microcirculation. The maximum platelet inhibition occurred at a carnosic acid concentration of 31 μg/mL [36]. 

Mice with atopic dermatitis that were topically treated with carnosol showed a significant reduction in skin lesions compared with the control animals [26]. Atopic dermatitis is a chronic inflammation of the skin characterized by the presence of eczematous and pruritic lesions related to several factors, such as inflammatory cells, cytokines, and enzymes (e.g., STAT3, iNOS, and COX-2) [37]. The lymph node weight and size were significantly increased, which is associated with a high immunoglobulin E production [38]. Carnosol was able to reduce the amounts of immunoglobulin E, neutrophils, and inflammatory cytokines (TNF-α and IL-1β) in the blood of mice. Histological sections of the ear and dorsum showed that the skin of the animals treated with carnosol was thinner and showed less infiltration of inflammatory cells and fewer mast cells compared with that of mice in the control group. Carnosol was able to reduce the expression of the enzymes iNOS and COX-2. Although it did not affect the expression of STAT3, the metabolite was able to inhibit the activity of this enzyme in in vitro assays; different concentrations of carnosol (1.2 and 5 μM) reduced NO production in LPS-treated RAW 264.7 cells [26]. 

*R. officinalis* did not show significant anti-inflammatory effects in induced dermatitis. In a study, the effect of extracts of marigold and rosemary on irritant contact dermatitis induced by sodium lauryl sulfate was evaluated in healthy human volunteers. Both extracts were incorporated into the base cream DAC (Deutscher Arzneimittel Codex = German Pharmaceutical Codex) at a 5% concentration. The effect of this formulation on irritant contact dermatitis was evaluated visually using bioengineering methods (e.g., chromametry and tewametry). These extracts were shown to have no anti-inflammatory effect on existing dermatitis; however, when applied simultaneously with the irritant, the inflammatory process was reduced, indicating that they exerted a protective effect against the development of induced dermatitis [54]. 

Evaluation of the topical anti-inflammatory effects of the extracts of rosemary leaves prepared with solvents of increasing polarity in vivo was performed using the croton oil-induced ear edema test. The extract obtained with n-hexane and chloroform exhibited important dose-dependent anti-inflammatory activity, whereas that obtained with methanol had a weak anti-inflammatory effect. The extracts obtained with chloroform ((ID_50_ = 83 μg/cm^2^) and n-hexane (ID_50_ = 265 μg/cm^2^) showed an anti-inflammatory activity similar to that of indomethacin (ID_50_ = 93 μg/cm^2^) (used as reference, with a discrete effect) [24].

### 3.2. Skin Cancer

The effects of *R. officinalis* hydroalcoholic extract were tested on human melanoma A375 cells; the extract resulted in a dose-dependent reduction of human melanoma cell proliferation. Mutations in melanocytes are attributed to the excessive exposure of the skin to sunlight and induce the development of melanomas [55,56,57]. The cell cycle proliferation was inhibited in vitro because of the cytotoxic and cytostatic activity of the hydroalcoholic extract [39]. When a mouse model of skin cancer induced by 7,12-dimethylbenz[a]anthracene was treated with a 500 or 1000 mg/kg oral dose of *R. officinalis* hydroalcoholic extract for a period of 15 days, it led to a decrease in the number, diameter, weight, and incidence of tumors and an increase in the latency period [40].

Rosmarinic acid was shown to exhibit chemopreventive activity against 7,12-dimethylbenz[a]anthracene-induced skin cancer; this was attributed to its anti-lipid peroxidation potential and its ability to modulate the detoxification cascade and expression patterns of p53, bcl-2, caspase-3, and caspase-9 [41]. Carnosic acid was shown to have an important protective role against melanoma. This secondary metabolite inhibited the proliferation and adhesion of B16F10 melanoma cells in a dose-dependent manner through the inhibition of the expression of cell migration markers (MMP-9, TIMP-1, uPA, and VCAM-1) and phosphorylation of signaling molecules (Akt, FAK, and Sr) [42].

### 3.3. Wound Healing

Healing is a complex dynamic process that results in the restoration of the anatomical barriers of the skin that may have been compromised by diseases or burns [58]. Diabetes was induced in male BALB/c mice by the intraperitoneal injection of alloxan [1]. After confirmation of hyperglycemia, incisions that were 4 mm in diameter were made on the backs of the mice and the mice were allocated to one of four treatments: control: vehicle, aqueous extract, and essential oil. The male BALB/c mice in the essential oil group showed healing, angiogenesis, and improvements in granulation tissue at several stages compared with those in the aqueous extract group [1]. Another study using diabetic rats topically treated with oil extracted from *R. officinalis* reported accelerated wound healing in both diabetic and nondiabetic animals [43].

Another study that explored the healing potential of rosemary was performed on 63 Wistar rats allocated to one of three treatments: control; 2% *R. officinalis* cream; and 4% *R. officinalis* cream. Wounds were made on each rat, and a solution containing *Candida albicans* was applied onto these wounds. The results showed that wound healing in the 4% *R. officinalis* cream group was faster than that in the other groups [44].

### 3.4. Skin Flap Survival

Skin flaps are used in the reconstruction of soft tissues and large wound defects. This technique has been employed in plastic surgery, and its efficacy is dependent on the location of the wound and extent of the defect [59,60].

One study compared animals allocated to three treatment groups: group I (control, the tissue was cleaned only with saline solution); group II (application of the essential oil on the tissue twice per day for 1 week following skin evaluation); and group III (application of rosemary oil before and after evaluation and at the end of the week, 30 min before the surgery). The survival rates were 29%, 59%, and 67% for groups I, II, and III, respectively. Compared with the control (group I), tissue necrosis was significantly lower and tissue viability was significantly higher in groups II and III. Topical application of the oil in the week before the surgery increased the tissue survival rate (higher in group III than in group II). The study reported antioxidant, anti-inflammatory, and vasodilatory activities of the oil as factors for the increased tissue survival [45].

### 3.5. Transdermal Effects

Transdermal drug delivery means that the drug is able to reach systemic circulation when administered topically. Drugs can penetrate the skin through three different pathways: transappendageal (when the drug permeates through hair follicles and through sweat and sebaceous glands); paracellular (when the drug passes through the intercellular space); and transcellular (when the drug passes through cells) [61]. The antinociceptive effects of the essential oil extracted from *R. officinalis* were analyzed following the incorporation of three concentrations (0.1%, 0.5%, and 1%) of the essential oil in a 1% diclofenac sodium gel. Two in vivo tests, the tail flick tests and formalin test, were performed to compare the antinociceptive effects of the gel base (control), gel with 1% diclofenac, and gel with 1% diclofenac plus 0.1%, 0.5%, and 1% rosemary oil. In both the in vivo assays, the 0.1% and 0.5% concentrations resulted in no significant antinociceptive effects whereas the essential oil concentration of 1% (in the diclofenac sodium gel) resulted in a reduction in pain. The study demonstrated that the essential oil is rich in monoterpenoids, especially 1,8-cineole, which are capable of promoting cutaneous absorption [46].

### 3.6. Antifungal Treatment

Dermatophytes are the most common agents causing topical mycoses [62]. The World Health Organization estimates that 20% of the global population is affected by dermatomycoses [63]; the prevalence of these diseases tends to increase with age and is dependent on the climate and location [64]. *R. officinalis* was reported to be active against dermatophytes in vivo [22].

The antifungal activity of rosemary essential oil was tested against *Candida albicans*, *Candida dubliniensis*, *Candida parapsilosis*, and *Candida krusei* [47]. Such dermatophytes are the most common agents causing topical mycoses [62]. It was found that an oil concentration of 8% was capable of inhibiting the growth of *Candida* sp. A similar study evaluated the effect of *R. officinalis* hydroalcoholic extract against two dermatophytes, *Microsporum gypseum* and *Trichophyton rubrum*, and showed that a concentration of 10% *R. officinalis* extract was responsible for 86% inhibition of fungal growth [48].

## 4. Cosmetic Properties

### 4.1. Ginoid Lipodystrophy (GLD, Cellulite)

Most postadolescent women have cellulite [65,66]. Cellulite is manifested by topographic disorders of subcutaneous tissue, such as nodules, edema, and abnormal fibrosis [67].

It is believed that gynoid lipodystrophy (GLD) is a chronic inflammatory process in which adipocyte malfunction causes a higher content of altered lipids to be retained. This increases the cellular volume and compromises blood circulation and the normal physiological state of pregnancy, after which the mother retains a higher content of lipids to guarantee caloric reserves [68].

A cream with extracts of three plants (*Zanthoxylum clava-herculis* (containing magnoflorine and laurifoline), *Annona squamosa* (containing squamosin and kaurenoic acid), and *Rosmarinus officinalis* (containing carnosic acid)) was examined for its effects on cellulite in a single-blind, randomized controlled study of 44 women between 18 and 59 years or age with mild-to-severe cellulite. The formulation led to an improvement in the appearance of the cellulite [36].

### 4.2. Alopecia

Alopecia is characterized by the loss of some or all hair and is classified as a chronic dermatological disorder [69]. The prevalence of alopecia has increased owing to stress and diet-related factors [70]. Excess testosterone in the blood capillaries is significantly associated with this condition; as such, antiandrogenic agents have been reported to reduce hair loss [49].

C57BL/6 mice with testosterone-induced alopecia were treated topically with hydroalcoholic extracts of rosemary (2 mg/day/animal) and showed a significant increase in hair growth after the 16th day of treatment compared with those in the control [49]. A hydroalcoholic extract was tested in vitro for the evaluation of 5αR enzyme activity and showed strong inhibition of the binding of dihydrotestosterone (DHT) to its receptor. An in vitro assay in human prostate LNCaP cells indicated that 12-methoxy-sarcosalic acid had a key role in the inhibition of the 5αR enzyme and DHT/receptor binding [49].

### 4.3. Antiaging

Aging is a skin process that occurs owing to intrinsic and extrinsic factors. Intrinsic factors act at the cellular level, whereas extrinsic factors are governed by human behavior, such as chronic exposure to the sun, poor nutrition, smoking, and excessive alcohol consumption [71]. These internal and external agents lead to the production of reactive oxygen species (ROS) [72]; when ROS levels exceed the cell’s neutralization capacity, damage to cell constituents occurs, ultimately leading to cell death [73].

A new compound was isolated from the hydrophilic fraction of an aqueous methanol extract and was named Rosm1. This biomolecule had a strong antioxidant capacity to neutralize ROS, similar to vitamin E, and was able to inhibit free radical-mediated reactions in vivo and in vitro, protecting lipids and cell constituents from oxidative stress [8].

Lipid nanoparticles have been used to increase the cutaneous permeation of drugs [74,75]. These nanoparticles were loaded with *R. officinalis* essential oil incorporated into a gel and an in vivo test was performed to assess the moisturizing ability of this formulation and any increase in elasticity. The gel containing nanoparticles loaded with rosemary oil showed a greater capacity to hydrate and increase of the elasticity of skin compared with the gel containing free essential oil [50].

Rosmarinic acid was encapsulated within chitosan microparticles. The release profile of the loaded particles was studied in two distinct media: water and coconut oil. A slower release profile observed in coconut oil was attributed to the lower solubility of the chitosan particles [76].

*R. officinalis* extract has strong antioxidant activity, which is mainly attributed to its phenolic compounds. Antioxidant activity is generally attributed to free radical scavenging, but secondary metabolites may play a biological role in the regulation of apoptosis, cell signal transduction, and xenobiotic metabolism in the liver [77].

### 4.4. Ultraviolet (UV) Protection

UVA radiation induces the production of ROS, and UVB is absorbed by molecules such as DNA and proteins, subsequently damaging the cells [78,79,80].

Rosemary extract was tested alone and in combination with citrus extract obtained from grapefruit in vivo and in vitro for its protective effects against UV irradiation [4]. In the in vitro cell viability measurement using the MTT (3-[4,5-dimethylthiazol-2-yl]-2,5-diphenyl tetrazolium bromide)assay, keratinocytes (HaCaT cells) were treated with the extracts separately and in combination. Then, the cells were exposed to UV radiation at intensities of 800 and 1200 J/m^2^. Both extracts increased the viability of the keratinocytes. The effects of a 1:1 mixture of citrus extract and rosemary extract was tested on cells exposed to UV radiation; this mixture showed superior effects on the increase of cell viability compared to those of either extract alone, indicating that the polyphenolic compounds of each extract have different targets for cell protection [4]. 

When irradiated with UV at a dose of 800 J/m^2^, the citrus and rosemary extracts (50 µg/mL each) showed 40% and 13% protection against UV light, respectively. When combined (100 µg/mL), 70% protection was observed. The synergistic effect was even greater when 1200 J/m^2^ of radiation was used. The extracts also had the ability to decrease the formation of free radicals during UV exposure. The study also showed that, when used in combination, the extracts were able to protect DNA from damage caused by the formation of free radicals. Oral administration of the combined citrus and *R. officinalis* extracts over the course of 8 weeks increased the UV dose required to induce erythema in the skin [4].

When the skin is injured by sun exposure, there is a decrease in type I collagen synthesis and excessive degradation by enzymes called metalloproteinases (MMPs). It was demonstrated that rosemary extract inhibits UV-induced metalloproteinase-1, indicating that it may reduce the skin damage caused by sunlight [51].

## 5. Other Studies

The impact of rosmarinic, ursolic, and oleanolic acids on the stability of multiple water/oil/water (W/O/W) emulsions has been studied. These acids did not have any effect on the interfacial tension when used as surfactants but were able to improve the stability of these emulsions for a short period of time. The authors concluded that rosemary extract contained active compounds with cosmetic potential owing to their various biological activities but that they could also be used as stabilizers, and favored the formation of W/O/W emulsions [52].

## 6. Conclusions and Future Perspectives

Rosemary extract contains a large variety of bioactive molecules with great therapeutic potential. These include triterpenes (e.g., ursolic and oleanolic acid), tricyclic diterpenes (e.g., carnosic acid and carnosol), phenolic acids (e.g., caffeic acid and rosmarinic acid), and essential oils. These secondary metabolites have been formulated in topical dosages. Topical administration strategies avoid first-pass metabolism, releasing the drug at the site of action and resulting in a lower risk of side effects. This strategy can be applied to improve the properties of cosmetic products (e.g., those that combat UV exposure, aging, and cellulite), owing to the anti-inflammatory activity and free radical-scavenging effects of *Rosmarinus officinalis*.

The use of nanoparticles in the development of new products for topical administration should be explored further, as they result in the more effective delivery of molecules to their site of action and increase treatment adherence. According to the published literature, rosemary has anti-inflammatory, antimicrobial, and antioxidant properties, which have been extensively reported in oral formulations (e.g., toothpaste or formulations to treat infections). The development of new formulations containing rosemary extracts should be encouraged, as their promise as topical agents is well established.

## Figures and Tables

**Table 1:** Results collected about *Rosmarinus officinalis* uses.

Topic	Results	Reference
**Anti-inflammatory activity**	Carnosic acid inhibit NO. Carnosic acid platelet was inhibited. Carnosol reduces atopic dermatitis. Rosemary extract showed anti-inflammatory activity similar to indomethacin.	[24,26,35,36,37,38]
**Skin cancer**	Rosemary extract reduces number, diameter, weight, and incidence of tumors and increases the latency period. Rosmarinic acid showed chameoprotective activity. Carnosic acid showed protective effect against melanoma.	[39,40,41,42]
**Wound healing**	Rosemary oil showed healing, angiogenesis, and improvements in granulation tissue. Rosemary oil accelerated healing wounds in diabetic and nondiabetic animals. Rosemary cream accelerated wound healing.	[1,43,44]
**Skin flaps survival**	Rosemary oil showed improvement in tissue survival and viability, and tissue necrosis was lower.	[45]
**Transdermal effects**	Monoterpertenes, presented in rosemary oil, promoted cutaneous absorption.	[46]
**Antifungal activity**	Rosemary oil was capable of inhibiting *C. albicans* growth. Rosemary extract was responsible for inhibiting fungal growth.	[47,48]
**Ginoid lipodystrophy (GLD, cellulite)**	A cream with carnosic acid was responsible for an improvement of cellulitis appearance.	[36]
**Alopecia**	Rosemary extract showed a hair growth.	[49]
**Antiaging**	Roms1 has a strong antioxidant capacity. Rosemary essential oil nanoparticles showed greater capacity of hydration and improves the elasticity.	[8,50]
**Ultraviolet protection**	Rosemary and citrus extracts were able to improve cell protection against UV. Rosemary extract reduced skin damage caused by the sun.	[4,51]
**Other studies**	Secondary metabolites present in rosemary extract showed stabilizer emulsion properties.	[52]
